# Effects of Blood Products on Inflammatory Response in Endothelial Cells In Vitro

**DOI:** 10.1371/journal.pone.0033403

**Published:** 2012-03-16

**Authors:** Martin Urner, Inge K. Herrmann, Felix Buddeberg, Caroline Schuppli, Birgit Roth Z'graggen, Melanie Hasler, Urs Schanz, Manuela Mehr, Donat R. Spahn, Beatrice Beck Schimmer

**Affiliations:** 1 Institute of Anesthesiology, University Hospital Zurich, Zurich, Switzerland; 2 Institute of Physiology and Zurich Center for Integrative Human Physiology, University of Zurich, Zurich, Switzerland; 3 Clinic of Haematology, University Hospital Zurich, Zurich, Switzerland; University of Leuven, Rega Institute, Belgium

## Abstract

**Background:**

Transfusing blood products may induce inflammatory reactions within the vascular compartment potentially leading to a systemic inflammatory response. Experiments were designed to assess the inflammatory potential of different blood products in an endothelial cell-based in vitro model and to compare baseline levels of potentially activating substances in transfusion products.

**Methods:**

The inflammatory response from pre-activated (endotoxin-stimulated) and non-activated endothelial cells as well as neutrophil endothelial transmigration in response to packed red blood cells (PRBC), platelet concentrates (PC) and fresh frozen plasma (FFP) was determined. Baseline inflammatory mediator and lipid concentrations in blood products were evaluated.

**Results:**

Following incubation with all blood products, an increased inflammatory mediator release from endothelial cells was observed. Platelet concentrates, and to a lesser extent also FFP, caused the most pronounced response, which was accentuated in already pre-stimulated endothelial cells. Inflammatory response of endothelial cells as well as blood product-induced migration of neutrophils through the endothelium was in good agreement with the lipid content of the according blood product.

**Conclusion:**

Within the group of different blood transfusion products both PC and FFP have a high inflammatory potential with regard to activation of endothelial cells. Inflammation upon blood product exposure is strongly accentuated when endothelial cells are pre-injured. High lipid contents in the respective blood products goes along with an accentuated inflammatory reaction from endothelial cells.

## Introduction

Transfusion of blood products is routinely performed in hospitals on a daily basis. However, with an estimated frequency of approximately 1 in 5000, blood transfusion leads to an inflammatory state in recipients [1], [2]. Transfusion-induced inflammatory reactions manifests as systemic inflammatory response syndrome, potentially resulting in severe sepsis [3], failure of pulmonary function (transfusion related acute lung injury) [4], or multiple organ failure [5], [6], [7], [8]. Inflammatory responses have been reported to occur after transfusion of all types of blood components including whole blood, red blood cells (PRBC), platelet concentrates (PC), fresh frozen plasma (FFP); with FFP being the most implicated blood product [9].

It has been previously suggested that the severe inflammatory reaction within the vascular compartment requires two triggers: one trigger being the endothelial cell activation (as seen in surgery, infection, or trauma), followed by a second triggering action represented by the transfusion of blood products containing biologic response modifiers aggravating the inflammatory status [10], [11], [12], [13]. Hence the identification of priming and activating constituents in the blood products is considered to be of decisive importance in order to understand pathophysiological mechanisms of endothelial cell activation.

This work aims to assess the inflammatory response of endothelial cells upon exposure to various types of blood products such as PRBC, PC (pooled or collected by apheresis), and plasma (both solvent detergent and untreated FFP). Mimicking a state of endothelial activation *in vitro* using lipopolysaccharides (LPS), we investigated if the inflammatory response and migration of neutrophils through the endothelial cells layer is accentuated in pre-activated endothelial cells.

Soluble CD40 ligand (sCD40L) as well as lipids have been identified as potential biologically active mediators in stored blood products [14], [15], [16]. We therefore assessed baseline sCD40L and total lipid concentration of the blood products and compared the levels with the inflammatory response measured in endothelial cells, previously exposed to the respective blood product. Additionally, several other inflammatory mediators known to play a crucial role within the inflammatory orchestration, such as interleukins (IL-6; IL-8/CXCL8) [17], tissue factor (transforming-growth-factor-β, TGF-β) [18], and chemokines (monocyte chemoattractant protein-1, MCP-1/CCL2 [19]; chemokine ligand 1, CXCL1) [20] were assessed in relation to the blood product's storage age.

## Materials and Methods

The study design was approved by the local Ethic Committee of the University Hospital Zurich, Switzerland (StV 7-2007).

### Blood transfusion products

Aliquotes of 5 mL were taken from the blood products PRBC (n = 56), PC (n = 49; 25 pooled, 24 apheresis), solvent detergent (n = 20) or normal FFP (n = 50). After centrifugation at 2500 rpm, the supernatants were aliquoted and stored at −20°C (short-term storage). In the case of PC, samples were also taken at day 6 (beyond regular storage duration).

### Incubation of blood product supernatants with endothelial cells

#### Cell culture

Human microvascular endothelial cells (HMVEC) were cultured as described in [21].

#### Stimulation with endotoxin

Growth medium of HMVEC was replaced by EBM-2 medium with 1% FBS 24 hours before experiments. HMVEC were stimulated with LPS from Escherichia coli, serotype 055:B5 (Sigma, Buchs, Switzerland) in a concentration of 2 µg/ml in EBM-2/1% FBS for 6 hrs [22]. As control group, HMVECs were exposed to phosphate-buffered saline (PBS) in EBM-2/1% FBS instead of LPS.

#### Incubation with blood products

Before exposing the pre-stimulated cells to blood products, the LPS- (or PBS-) containing medium was removed and cells were washed once with PBS. Cells were exposed to samples of blood products (20 samples from each product chosen by random selection using matlabs ‘randi’ function) previously diluted 1∶2 in EBM-2/1% FBS, and supplemented with 12.5 I.E Heparin-Na (B/Braun Medical AG, Sempach, Switzerland). The control group was incubated with 1∶2 diluted PBS with EBM-2/1% FBS and 12.5 I.E Heparin-Na. All samples were incubated for 24 h at 37.0°C and 5% CO2. Afterwards, supernatants were collected and stored at −20°C until further testing by ELISA for IL-6, IL-8, and CXCL1.

### Neutrophil transmigration

#### Neutrophil isolation

Venous blood was obtained from healthy adult volunteers and anticoagulated with citrate dextrose solution (Sigma, St. Louis, MO). Neutrophils were isolated by gradient centrifugation over Ficoll-Paque (Amersham Pharmacia Biotech, Dubendorf, Switzerland) followed by 1% dextran sedimentation for 1 h to separate neutrophils from erythrocytes. Neutrophils were resuspended in Hank's balanced salt solution (HBSS; Life Technologies, Carlsbad, CA).

#### Transmigration assay

Transmigration experiments were carried out 96-well transmigration chambers (Millipore, Zug, Switzerland) where the upper compartment is separated from the lower one by a polycarbonate filtermembrane with pores of 3 µm in diameter. After culturing HMVEC in the upper compartment and prestimulation with LPS (or PBS), neutrophils (1.5×10^6^ cells/125 µl of medium) and blood product (125 µl) were placed in the upper compartment and incubated at 37°C for 2 h. Transmigrated neutrophils were then quantified using a glucuronidase assay [23]. In short, cells in the lower compartment were lysed in 1% Triton X-100, and glucuronidase enzymatic activity was measured by using *p*-nitrophenyl-glucuronide (Sigma-Aldrich Chemie, St. Louis, MO) as a substrate.

### Cytokine concentration

Supernatant samples were tested by enzyme-linked immunosorbent assays according to the manufacturer's protocol for the following inflammatory mediators: sCD40L, IL-6, IL-8, TGF-β, MCP-1, and CXCL1 (R&D Systems Europe Ltd, Abingdon, UK). Optical density was determined at 450 nm and results were given in pg/ml.

### Lipid concentration

For the quantitative determination of the total lipid concentration in the samples, we used the sulfo-phospho-vanillin colorimetric method [24]. All reagents were purchased from Sigma-Aldrich (Buchs, Switzerland). Glass tubes were purchased from Vitaris AG (Baar, Switzerland).

### Statistical analysis

Statistical analyses were performed with OriginPro 8G (Origin Lab, Northampton, MA) and SPSS 17 (SPSS Inc., Chicago, IL). Boxplot figures show medians and quartiles. Whiskers represent 5% and 95% confidence intervals, * represent 1% and 99% confidence intervals. Linear regression was used to assess inflammatory response in endothelial cells and transendothelial migration of neutrophils with regard to blood product exposure (dependent variables: interleukin-6, IL-8, and CXCL1 concentrations, migrated neutrophils; independent variables: type of blood product; **Tables S1, S2, S3, S4**). Pearson correlation coefficients were calculated to determine the correlation of inflammatory response in endothelial cells and transendothelial migration of neutrophils with baseline sCD40L and total lipid concentration in blood products (**Tables 1** **and** **2**). Spearman's rank correlation coefficients were calculated to reveal storage-dependent changes in inflammatory mediator levels or lipid levels of PRBC or PC (**Tables S5 and S6**).

**Table 1:** Correlation of inflammatory mediator release in endothelial cells with baseline sCD40 and lipid concentration in blood products.

		lipids	sCD40L
**IL-6**	Pearson correlation	**0.269**	−0.112
	Significance	<0.001 *	0.119
**IL-8**	Pearson correlation	0.075	−0.124
	Significance	0.299	0.085
**CXCL-1**	Pearson correlation	**0.146**	−0.131
	Significance	0.042 *	0.069

N = 194.

IL-6: interleukin-6; IL-8: interleukin-8; CXCL1: chemokine ligand 1; sCD40L: soluble CD40 ligand.

**Table 2:** Correlation of transendothelial migration of neutrophils with baseline sCD40L and lipid concentration in blood products.

		Lipids	CD40
**Transmigration**	Pearson Korr.	**0.707**	0.122
	Sig.	<0.001 *	0.343

N = 62.

sCD40L: soluble CD40 ligand; Transmigration = Transmigration was estimated measuring glucuronidase enzymatic activity in the lower compartment of the transmigration chamber.

## Results

### Inflammatory response of unstimulated and pre-stimulated endothelial cells upon incubation with different blood products

**Figure 1:**
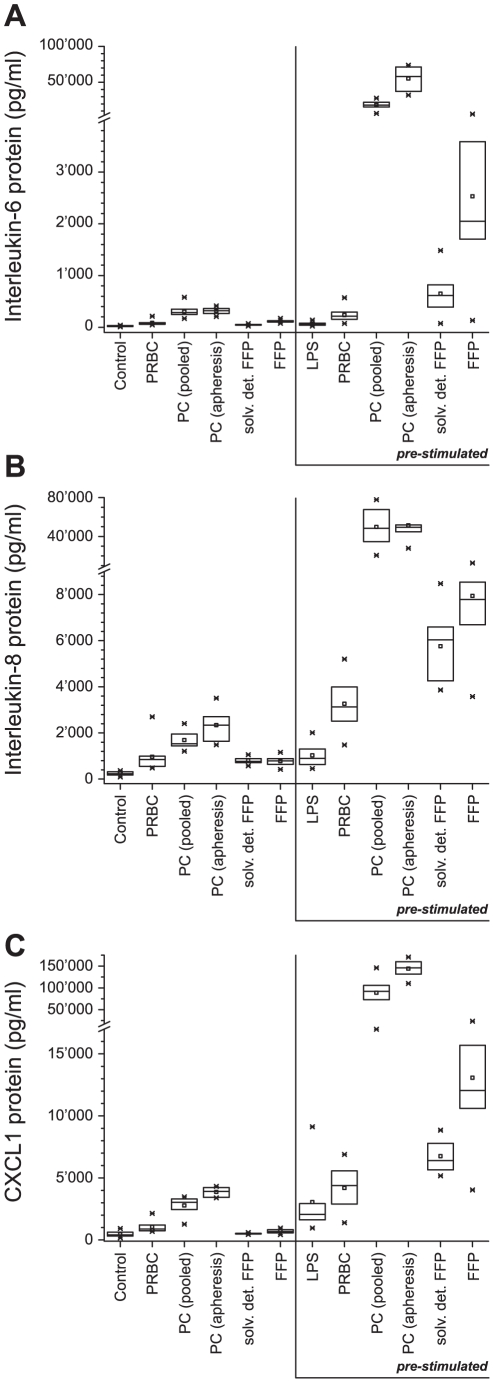
Inflammatory reaction of endothelial cells in response to exposure to different blood products. Concentrations of interleukin-6, IL-6 (A), interleukin-8 (IL-8) (B) as well as chemokine ligand 1 CXCL1 (C) in supernatants of human microvascular endothelial cells (HMVEC). HMVEC were stimulated with lipopolysaccharide (LPS) 2 µg/ml for 6 h (pre-stimulated) or not (unstimulated), followed by an 24 h-incubation with the according blood products packed red blood cells (PRBC), platelet concentrates (PC), pooled or apheresis, and solvent detergent or untreated fresh frozen plasma (FFP). Supernatants were collected and IL-6, IL8 and CXCL1 were determined. Results are shown as medians with interquartile ranges.

The inflammatory potential of PRBC, PC and FFP was quantified measuring the inflammatory mediator release from human microvascular endothelial cells in response to exposure to different blood transfusion products. As markers of the inflammatory response, *de-novo* protein synthesis of IL-6, IL-8, and CXCL1 was measured (**Fig. 1**).

In unstimulated endothelial cells, increased IL-6, IL-8 and CXCL1 protein levels were found after incubation with different blood products (at least 2.3-fold (PRBC), 6-fold (PC), 1.5-fold (FFP), increase relative to control, p<0.001).

Using LPS-stimulated endothelial cells, the cytokine release was much more pronounced compared to unstimulated cells (p<0.001). Incubation with PC (both pooled and apheresis) induced the most accentuated increase in production of mediators (at least 30-fold increase in cytokine levels relative to LPS) (p<0.001, results of the linear regressions in **Tables S1, S2, S3**). In supernatants from endothelial cells incubated with untreated FFP, higher cytokine concentrations were found compared to solvent detergent FFP (2.5-fold higher average cytokine levels in untreated FFP), however, cytokine concentrations remained well below the expression levels observed for PC (**Tables S1, S2, S3**).

### Neutrophil transmigration through non-activated and activated endothelial cell layers

**Figure 2:**
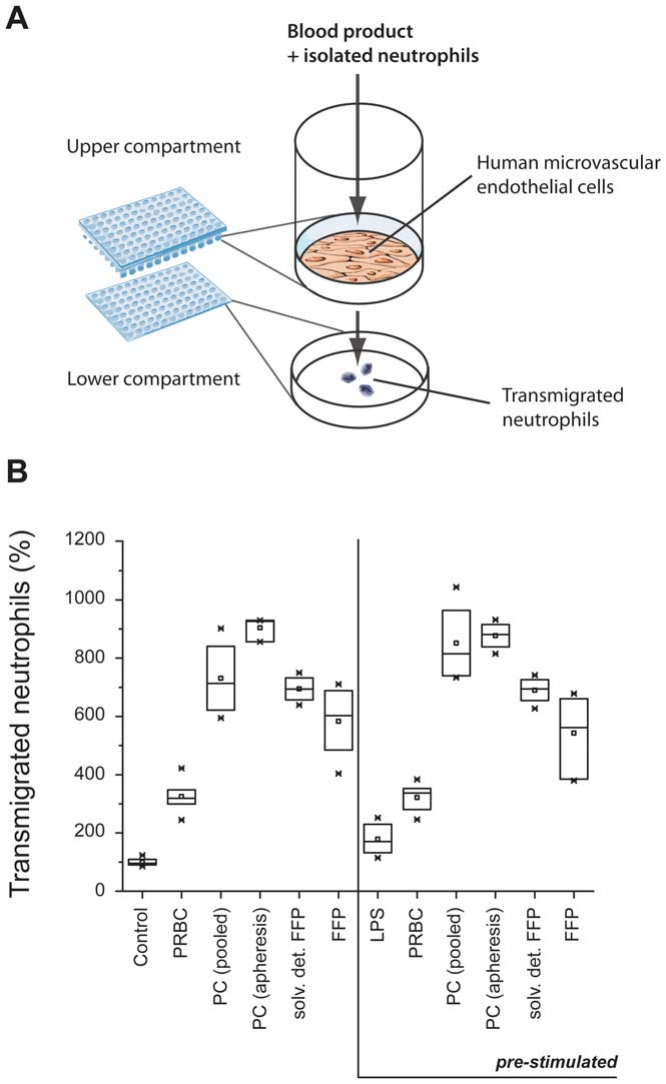
Neutrophil transmigration through an endothelial cell layer after exposure to blood products. Transmigration of neutrophils through unstimulated and pre-stimulated monolayer of human microvascular endothelial cells (HMVEC) (A). HMVEC were stimulated with lipopolysaccharide (LPS) 2 µg/ml for 6 h (pre-stimulated) or not (unstimulated), followed by a 2 h-incubation with the according supernatant of previously centrifuged blood products such as packed red blood cells (PRBC), platelet concentrates (PC), pooled or apheretic, and solvent detergent or untreated fresh frozen plasma (FFP) together with 1.5×10^6^ neutrophils. Transmigrated neutrophils were measured using a glucuronidase assay (B). Results are shown as medians with interquartile ranges.

Transmigration of human neutrophils through an unstimulated and LPS-pre-stimulated endothelial cell layer was assessed (experimental setup **Fig. 2A**): Similar to the production of inflammatory mediators upon exposure to blood products, the number of transmigrated neutrophils was most accentuated for PC and FFP (**Fig. 2B**). However, no significant differences in transmigration were observed between non-stimulated and pre-stimulated endothelial cells for all blood products, except for PC pooled (p = 0.04, **Table S4**).

### Differences in sCD40L and total lipid concentration among different blood products

A detailed characterization of the used blood products regarding the inflammatory potential is a pre-requisite regarding the evaluation and interpretation of the inflammatory response upon incubation with endothelial cells in the vascular compartment. To obtain a first overview on baseline characteristics of the different blood products, concentrations of sCD40L protein as a highly proinflammatory cytokine were measured and compared. Furthermore total lipid concentration for each blood products was assessed.

**Figure 3:**
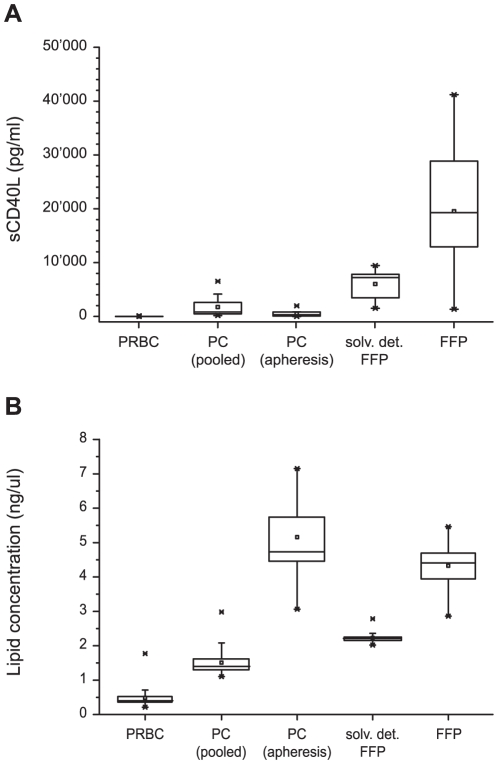
sCD40L protein and total lipid concentrations in different blood products. Concentration of soluble CD40 ligand (sCD40L) (A) and total lipids (B) in packed red blood cells (PRBC), platelet concentrates (pooled and apheresis), solvent detergent or untreated fresh frozen plasma (FFP). Blood products were collected and centrifuged at 1500 rpm. Supernatants were analyzed with enzyme-linked immunosorbent assays for sCD40L, concentrations of lipids were determined using the sulfo-vanillin colorimetric method. Results are shown as medians with interquartile ranges.

While in PRBC and PC only marginal expression of sCD40L was found, solvent detergent fresh-frozen plasma showed a fiftyfold, and untreated FFP hundred times higher sCD40L concentrations in comparison to PRBC and PC (**Fig. 3A**). Soluble CD40L concentrations measured in apheresis PC were not significantly lower than in pooled PC.

Regarding total lipid levels, the lowest concentrations were measured in PRBC (median: 0.4 ng/ul), while in apheresis PC and FFP much higher lipid concentrations (medians: 4.7 ng/ul and 4.4 ng/ul, respectively) were observed (**Fig. 3B**). A remarkable difference between lipid concentrations of pooled PC and apheresis PC was found: The median lipid concentrations measured in apheresis PC (median: 4.7 ng/ul) was about three times higher than in pooled PC (median: 1.4 ng/ul). Untreated FFP showed a lipid concentration of 4.4 ng/ul (median value), whereas in solvent detergent FFP, lipid concentration (median value 2.2 ng/ul) was only half of the concentration measured in normal FFP.

### Effect of storage time on the concentration of potentially activating substances present in the blood products

#### Storage-dependent increase of cytokine concentrations in packed red blood cells

To evaluate baseline levels of inflammatory cytokines in blood products, inflammatory mediators were determined as function of the storage time of the PRBC (8 days–42 days). In addition to sCD40L, protein levels of IL-6, IL-8, TGF-β, MCP-1, and CXCL1 were assessed. IL-6, IL-8, and TGF-β protein concentrations remained below the detection limits over the whole storage period of 42 days. For sCD40L, MCP-1 and CXCL1 concentrations, an increase with storage age was observed (**Figure S1, Table S5**). The increase in protein levels is most pronounced at the end of the storage period after day 36.

#### No storage-dependent change in cytokine concentrations in platelet concentrates

The same inflammatory mediators were also determined for PC as function of storage time. IL-6, IL-8, and CXCL1 levels remained below the detectable range over the measured time period up to 6 days. Soluble CD40L, MCP-1, and TGF-β protein concentrations did not change in an age dependent manner during the storage period of six days (**Figure S2, Table S5**).

#### Assessment of lipid concentrations in packed red blood cells and platelet concentrates

**Figure 4:**
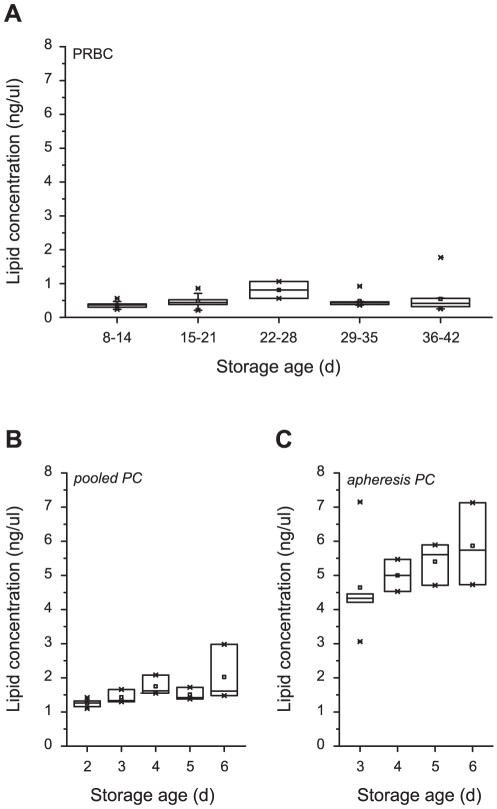
Storage-dependency of lipid concentration in blood products. Storage-dependent concentration of lipids in packed red blood cells (PRBC) (A), and in pooled (B) and apheresis platelet concentrates (C). Concentrations of lipids were determined using the sulfo-vanillin colorimetric method. Results are shown as medians with interquartile ranges.

To reveal potential storage-dependent changes in lipid content (breakdown products of membrane lipids), lipid concentrations were assessed in PRBC and PC. In PRBC no significant changes were observed over a storage period of up to 42 days (Spearman's rank correlation coefficient: 0.214; p-value: 0.180; **Fig. 4** and **Table S6**). Regarding pooled and apheresis PC, a storage-age dependent increase in lipid concentrations after 6 days of storage was found (**Fig. 4** and **Table S6**). The storage age had no significant influence on neutrophil transmigration (**Table S7**).

### Correlation of baseline sCD40L and total lipid levels in blood products on the inflammatory response in endothelial cells and transendothelial migration

Baseline levels of potential biologic response modifiers such as sCD40L and total lipids were analyzed regarding their influence on *de novo* synthesis of inflammatory mediators in endothelial cells. No correlation with baseline sCD40L concentration in blood products was found. The release of IL-6 and CXCL-1 protein was correlated with baseline total lipid concentration in blood products (**Table 1** and **Table S8**). This could not be shown for production of IL-8 protein. Also transendothelial migration was in good correlation with total lipid levels in blood products, but not with baseline sCD40L concentrations (**Table 2** and **Table S9**).

## Discussion

In the present work, we were able to show that transfusion-approved PC and to a lesser extent FFP provoke a strong release of inflammatory mediators in endothelial cells, which is even more accentuated in LPS-prestimulated endothelial cells. Secretion of inflammatory mediator as well as neutrophilic endothelial transmigration was in good agreement with the lipid content of the according blood products.

### Inflammatory mediator release in pre-activated endothelial cells in response to blood product exposure

While PRBC lead only to a minor increase in mediator expression, PC and to a lesser extent FFP induced a strong release of inflammatory mediators in endothelial cells. This inflammatory response was even more pronounced when cells were previously exposed to moderate doses of LPS (‘activated endothelium’). Such pre-activation of the endothelium may have severe consequences: blood products can induce an extra production of inflammatory mediators in activated compared to non-activated endothelial cells.

Differences in inflammatory potential among the different blood components were found regarding stimulation of the endothelial cells. Plasma-rich components and platelets appear to be particularly active with regard to the endothelial inflammatory mediator secretion, which in turn raises questions about biological response modifiers [25] present in the respective blood components (baseline levels) and their storage time dependent accumulation. Although variations in inflammatory response were observed for different bags of the same blood product, the importance was clearly secondary to the observed differences among transfusion products.

### Differences in mediator levels among different blood products and storage-dependent accumulation of biologically active mediators

No time-dependent changes in cytokine concentration up to 35 days of storage were found in leukocyte-reduced PRBC, however, after 35 days, a slight increase in concentration of sCD40L, MCP-1, and CXCL1 was measured. In leukocyte-reduced PC we did not find any change in cytokine concentration over storage time. This is in accordance with other studies, showing that accumulation of cytokines in blood products could be prevented by pre-storage leukocyte-reduction [26], [27], [28], [29], [30]. Interestingly, cytokine concentrations vary widely among different units of leukocyte-depleted products, suggesting that the cytokines originate from time points before leukocyte-reduction. Indeed, Chudziak et al. [31] found a strong correlation between cytokine concentration and time of leukocyte-reduction in blood products. In conclusion, high cytokine concentrations seem to be rather a result of late leukocyte removal during blood product manufacturing than of prolonged storage time.

With regard to lipid concentration no increase was observed for PRBC between 8 and 42 days. While the concentration was low in the pooled PC products and remained at this level for 6 days, higher concentrations of lipids were measured in the apheresis PC with an increase over time. If this observation is due to the technique of cell separation needs further investigation. A previous *in vitro* study has found impaired function of PC after a storage time exceeding 7 days [32]. Additionally, Schrezenmeier et al mentioned in a recent review article, that several factors might influence the quality of PC such as extent of cell separation (residual leukocytes), plasma content or additive solution which in turn might affect also lipid concentration of PC [33].

### PMN transmigration is correlated to inflammatory response but independent on pre-activation of endothelial cells

Effector cell infiltration and migration through the endothelium orchestrated by proinflammatory mediators and chemoattractants secreted from endothelial cells is a crucial step in the evolvement of tissue damage [34]. We quantitatively assessed the number of polymorphonuclear neutrophils transmigrating through an endothelial cell monolayer in the presence of different blood products. Transmigration might reflect an active process of the neutrophils triggered by the release of chemoattractant inflammatory mediators, secreted by already transmigrated neutrophils. Alternatively, a passive transmigration due to a neutrophil-induced destruction of the endothelial monolayer with increased permeability might be observed. Indeed, the number of transmigrated neutrophils was in good agreement with the observed relative inflammatory mediator secretion: neutrophil migration was most accentuated in the presence of PC and FFP. Interestingly, neutrophil net migration was, however, unaffected by the LPS priming of the endothelial cell layer. This stays in contrast to the amplified inflammatory cytokine expression observed for pre-activated endothelial cells.

### Can the ‘inflammatory response pattern’ be predicted based on concentration of sCD40L and/or total lipid?

As differences in sCD40L and lipid concentrations among the different types of blood products were observed, inflammatory response of endothelial cells as well as transendothelial effector cell migration were compared to the measured baseline sCD40L and total lipid concentrations in blood products. An identical pattern of lipid levels and inflammatory mediator release was found: while the lipid levels and the observed inflammatory response in PRBC were moderate, the lipid as well as the inflammatory mediator levels reached their maximum for PC products. In agreement with our results, a recent study showed that lysophosphatidylcholines in PC might contribute to pulmonary inflammation [35]. Also nonpolar lipids in PRBC are thought to play a certain role in the inflammation process [36].

### Limitations of the used experimental approach

In contrast to the raise in proinflammatory mediators after incubation with blood products measured in the present endothelial cell model, only few patients develop severe inflammatory reactions upon blood product administration. It is believed that in patients, there is a complex balance between pro- and anti-inflammatory factors which ultimately determines the reaction of the patient in response to blood product transfusion wherein the state of pre-activation in endothelial cells plays an important role. However, in vitro models may only replicate a specific part of such complex reactions. In addition, in this in vitro model, endothelial cells were studied under static and isolated conditions which do not accurately mimic the dynamic response of a whole organism to blood transfusion. In the present study, experiments were performed using blood products ready for transfusion received from the hospital's blood bank. To the best of our knowledge, unprocessed fresh blood is not readily used for transfusion and was therefore not considered to be of relevance in this study.

Beside stimulation using lipopolysaccharides there are several other potent activators (such as TNF-α or interleukin-1 beta) of endothelial cells [37]. The use of lipopolysaccharides to provoke the release of proinflammatory mediators is very well established and has been described in cell and animal models, as well as in human volunteers [38], [39], [40].

### Conclusion and outlook

This work compares for the first time the inflammatory response in endothelial cells upon exposure to all important blood products in the same setting. Blood products are able to provoke an inflammatory response in endothelial cells, especially when cells are already pre-activated. Platelet concentrates appear to be more potent than FFP as trigger for the inflammatory reaction within the vascular compartment.

As the inflammatory reaction most likely goes along with lipid content of the blood products, further investigations are now needed to quantitatively analyze the lipid content in PC and FFP. Detailed elucidation of the lipid composition of blood product may crystallize factors to be removed from these two blood products leading to safer blood transfusion for the patients.

While several studies in the recent years have put major efforts in finding causative factors for blood product-induced inflammatory conditions in patients, the key compound responsible has not yet been identified. However, based on the above presented results, we hypothesize that lipids - causative or not - may represent a surrogate for the inflammatory potential of the blood product. This has now to be investigated in future in vivo experiments.

## Supporting Information

Figure S1 Storage-dependency of baseline cytokine concentration in packed red blood cells (PRBC).
(DOC)

Figure S2 Storage-dependency of cytokine concentration in platelet concentrates (PC).
(DOC)

Table S1 Influence of blood product exposure on interleukin-6 expression in endothelial cells.
(DOC)

Table S2 Influence of blood product exposure on interleukin-8 expression in endothelial cells.
(DOC)

Table S3 Influence of blood product exposure on CXCL1 expression in endothelial cells.
(DOC)

Table S4 Influence of blood product exposure on transendothelial migration of neutrophils.
(DOC)

Table S5 Spearman correlations of cytokine concentrations in packed red blood cells (PRBC) and platelets concentrates (PC) versus storage age.
(DOC)

Table S6 Spearman correlations of lipid concentration versus storage age in packed red blood cells (PRBC) and platelets concentrates (PC).
(DOC)

Table S7 Age-dependent assessment of neutrophil transmigration.
(DOC)

Table S8 Influence of baseline sCD40L and lipid concentration in blood products on inflammatory mediator release in endothelial cells.
(DOC)

Table S9 Correlation of baseline sCD40L and lipid concentration in blood products with transendothelial migration of neutrophils.
(DOC)
