# Supplementary material for: Effects of Blood Products on Inflammatory Response in Endothelial Cells In Vitro
Source: PLoS One. 2012 Mar 16;7(3):e33403. doi: 10.1371/journal.pone.0033403 (PMC3306413; doi:10.1371/journal.pone.0033403)

**Figure S1:** *Storage-dependency of baseline cytokine concentration in packed red blood cells (PRBC).*


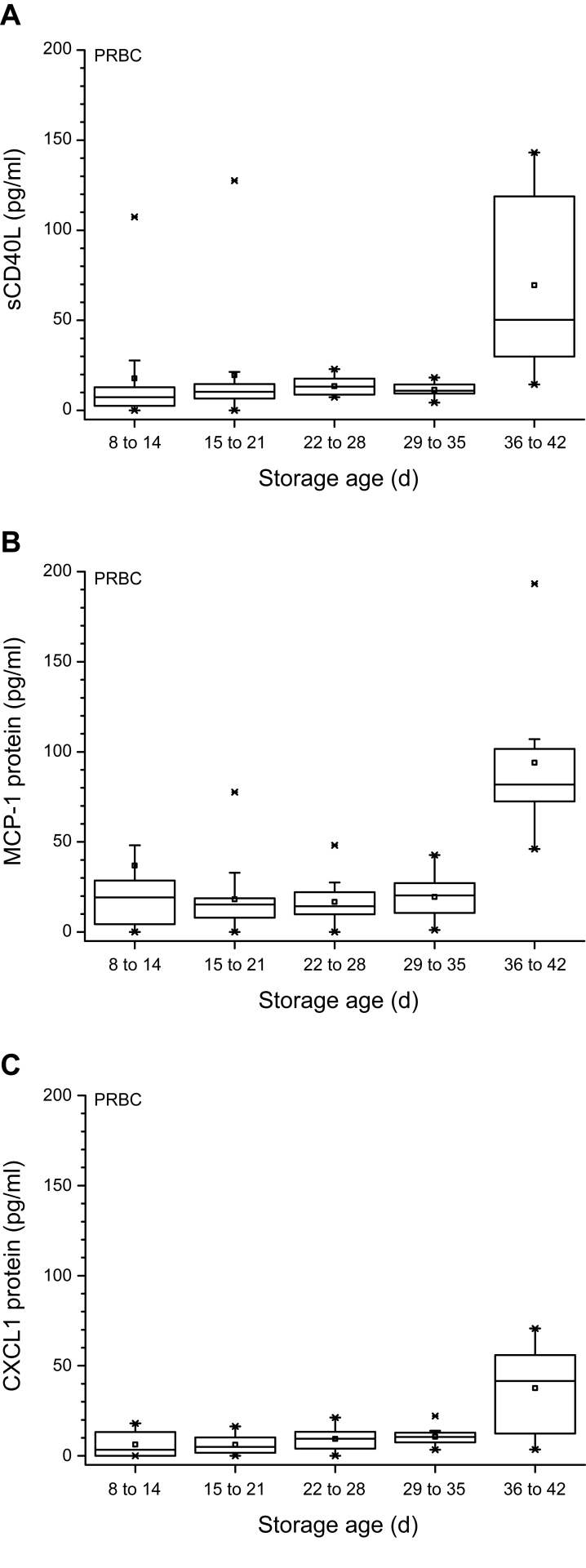

Supplement: Figure S1 — Storage-dependency of baseline cytokine concentration in packed red blood cells (PRBC). (DOC) [file pone.0033403.s001.doc]
