# Supplementary figures and images for: Effects of Blood Products on Inflammatory Response in Endothelial Cells In Vitro
Source: PLoS One. 2012 Mar 16;7(3):e33403. doi: 10.1371/journal.pone.0033403 (PMC3306413; doi:10.1371/journal.pone.0033403)

**Figure S2:** *Storage-dependency of cytokine concentration in platelet concentrates (PC).*


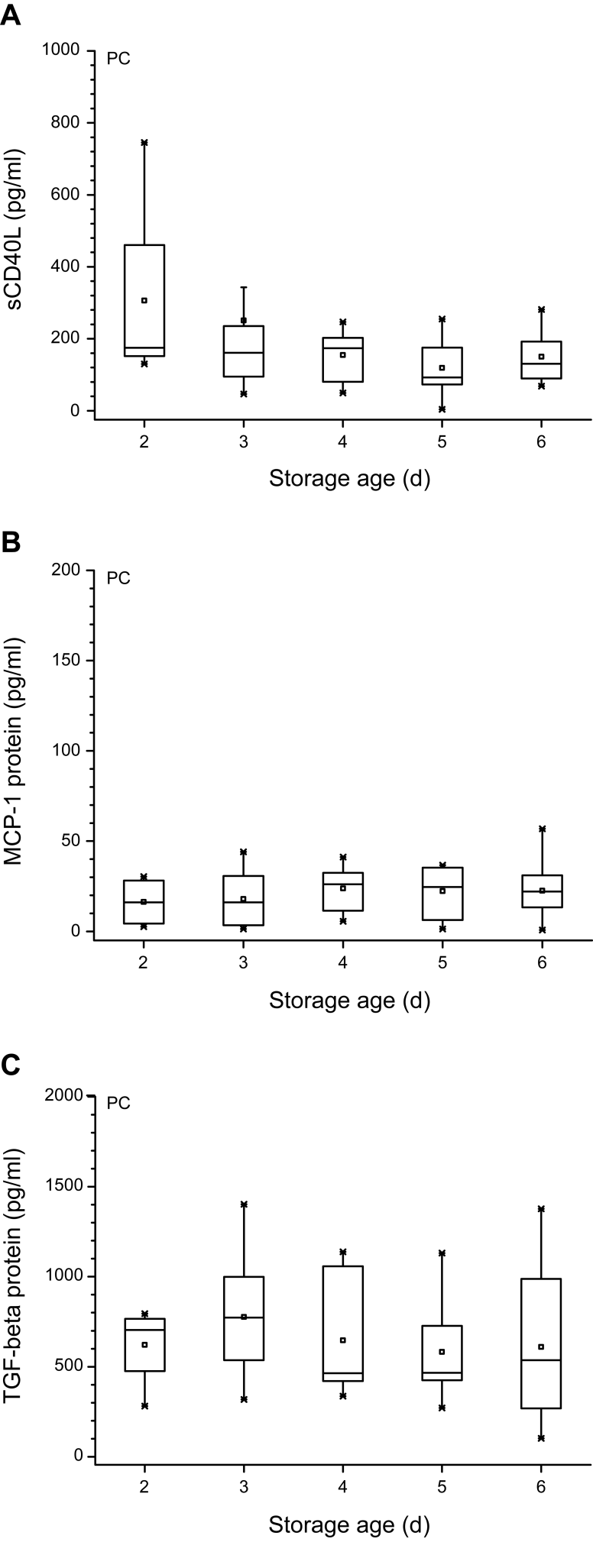

Supplement: Figure S2 — Storage-dependency of cytokine concentration in platelet concentrates (PC). (DOC) [file pone.0033403.s002.doc]
