# Supplementary material for: Effects of Blood Products on Inflammatory Response in Endothelial Cells In Vitro
Source: PLoS One. 2012 Mar 16;7(3):e33403. doi: 10.1371/journal.pone.0033403 (PMC3306413; doi:10.1371/journal.pone.0033403)
Supplement: Table S1 — Influence of blood product exposure on interleukin-6 expression in endothelial cells. (DOC) [file pone.0033403.s003.doc]

***Table S1.*** *Influence of blood product exposure on interleukin-6 expression in endothelial cells.*

| Independent Variable | Standardized Coefficients | Unstandardized Coefficients | 95% Confidence Interval for B | | Sig. |
| --- | --- | --- | --- | --- | --- |
|  | Beta | B | Lower Bound | Upper Bound |  |
| PRBC | -0.596 | -15285 | -19809 | -10761 | **<0.001** |
| PC apheresis | -0.348 | -15099 | -20562 | -9635 | **<0.001** |
| PC pooled | -0.506 | -15161 | -19899 | -10424 | **<0.001** |
| FFP | -0.520 | -15275 | -19984 | -10566 | **<0.001** |
| Solv. det. FFP | -0.522 | -15345 | -20054 | -10636 | **<0.001** |
| LPS * PRBC | 0.052 | 1703 | -909 | 4316 | 0.200 |
| LPS * PC apheresis | 0.849 | 50982 | 45924 | 56040 | **<0.001** |
| LPS * PC pooled | 0.365 | 14631 | 11348 | 17913 | **<0.001** |
| LPS * FFP | 0.062 | 2415 | -784 | 5614 | 0.138 |
| LPS * solv. det FFP | 0.015 | 602 | -2597 | 3801 | 0.711 |

R^2^: 0.820, N=200; dependent variable: interleukin-6 protein (pg/ml)

PRBC: packed red blood cells; PC: platelet concentrates; FFP: fresh frozen plasma; LPS: lipopolysaccharide
