# Supplementary material for: Effects of Blood Products on Inflammatory Response in Endothelial Cells In Vitro
Source: PLoS One. 2012 Mar 16;7(3):e33403. doi: 10.1371/journal.pone.0033403 (PMC3306413; doi:10.1371/journal.pone.0033403)
Supplement: Table S2 — Influence of blood product exposure on interleukin-8 expression in endothelial cells. (DOC) [file pone.0033403.s004.doc]

***Table S2.*** *Influence of blood product exposure on interleukin-8 expression in endothelial cells.*

| Independent Variable | Standardized Coefficients | Unstandardized Coefficients | 95% Confidence Interval for B | | Sig. |
| --- | --- | --- | --- | --- | --- |
|  | Beta | B | Lower Bound | Upper Bound |  |
| PRBC | -0.642 | -23687 | -30606 | -16767 | **<0.001** |
| PC apheresis | -0.360 | -22407 | -30763 | -14051 | **<0.001** |
| PC pooled | -0.535 | -23059 | -30305 | -15814 | **<0.001** |
| FFP | -0.568 | -23985 | -31187 | -16783 | **<0.001** |
| Solv. det. FFP | -0.567 | -23954 | -31155 | -16752 | **<0.001** |
| LPS * PRBC | 0.143 | 6757 | 2762 | 10752 | **<0.05** |
| LPS * PC apheresis | 0.550 | 47459 | 39722 | 55195 | **<0.001** |
| LPS * PC pooled | 0.752 | 43366 | 38347 | 48386 | **<0.001** |
| LPS * FFP | 0.127 | 7165 | 2272 | 12057 | **<0.05** |
| LPS * solv. det FFP | 0.088 | 4952 | 60 | 9845 | **<0.05** |

R^2^: 0.786, N=200; dependent variable: interleukin-8 protein (pg/ml)

PRBC: packed red blood cells; PC: platelet concentrates; FFP: fresh frozen plasma; LPS: lipopolysaccharide
