# Supplementary material for: Effects of Blood Products on Inflammatory Response in Endothelial Cells In Vitro
Source: PLoS One. 2012 Mar 16;7(3):e33403. doi: 10.1371/journal.pone.0033403 (PMC3306413; doi:10.1371/journal.pone.0033403)
Supplement: Table S3 — Influence of blood product exposure on CXCL1 expression in endothelial cells. (DOC) [file pone.0033403.s005.doc]

***Table S3.*** *Influence of blood product exposure on CXCL1 expression in endothelial cells.*

| Independent Variable | Standardized Coefficients | Unstandardized Coefficients | 95% Confidence Interval for B | | Sig. |
| --- | --- | --- | --- | --- | --- |
|  | Beta | B | Lower Bound | Upper Bound |  |
| PRBC | -0.727 | -62108 | -75542 | -48675 | **<0.001** |
| PC apheresis | -0.416 | -60047 | -76269 | -43824 | **<0.001** |
| PC pooled | -0.612 | -61058 | -75125 | -46991 | **<0.001** |
| FFP | -0.642 | -62786 | -76768 | -48804 | **<0.001** |
| Solv. det. FFP | -0.644 | -62971 | -76954 | -48989 | **<0.001** |
| LPS * PRBC | 0.114 | 12446 | 4690 | 20202 | **<0.05** |
| LPS * PC apheresis | 0.720 | 143783 | 128764 | 158802 | **<0.001** |
| LPS * PC pooled | 0.626 | 83532 | 73786 | 93278 | **<0.001** |
| LP S* FFP | 0.095 | 12376 | 2877 | 21875 | **<0.05** |
| LPS * solv. det FFP | 0.048 | 6233 | -3266 | 15732 | 0.197 |

R^2^: 0.857, N=200; dependent variable: CXCL1 protein (pg/ml)

PRBC: packed red blood cells; PC: platelet concentrates; FFP: fresh frozen plasma; LPS: lipopolysaccharide
