# Supplementary material for: Effects of Blood Products on Inflammatory Response in Endothelial Cells In Vitro
Source: PLoS One. 2012 Mar 16;7(3):e33403. doi: 10.1371/journal.pone.0033403 (PMC3306413; doi:10.1371/journal.pone.0033403)
Supplement: Table S4 — Influence of blood product exposure on transendothelial migration of neutrophils. (DOC) [file pone.0033403.s006.doc]

***Table S4.*** *Influence of blood product exposure on transendothelial migration of neutrophils.*

| Independent variables | Unstandardized Coefficient | | | Standardized Coefficients | Sig. |
| --- | --- | --- | --- | --- | --- |
|  | B | 5% CI | 95% CI | Beta |  |
| PRBC | 185.612 | 111.567 | 259.657 | .298 | **<0.001** |
| PC pooled | 591.809 | 498.148 | 685.469 | .718 | **<0.001** |
| PC apheresis | 764.840 | 660.125 | 869.556 | .874 | **<0.001** |
| solv. det. FFP | 554.907 | 480.862 | 628.952 | .892 | **<0.001** |
| FFP | 444.082 | 370.038 | 518.127 | .697 | **<0.001** |
| LPS * PRBC | -3.511 | -84.623 | 77.602 | -.004 | 0.931 |
| LPS * PC pooled | 120.638 | 5.928 | 235.348 | .107 | **0.04** |
| LPS * PC apheresis | -26.941 | -150.842 | 96.959 | -.024 | 0.665 |
| LPS * solv. det. FFP | -4.229 | -85.341 | 76.883 | -.005 | 0.917 |
| LPS * FFP | -40.914 | -124.873 | 43.045 | -.047 | 0.334 |

R^2^: 0.914; N=74; dependent variable: transendothelial migration (% of control)

PRBC: packed red blood cells; PC: platelet concentrate; FFP: fresh frozen plasma; LPS: lipopolysaccharide
