# Supplementary material for: Effects of Blood Products on Inflammatory Response in Endothelial Cells In Vitro
Source: PLoS One. 2012 Mar 16;7(3):e33403. doi: 10.1371/journal.pone.0033403 (PMC3306413; doi:10.1371/journal.pone.0033403)
Supplement: Table S5 — Spearman correlations of cytokine concentrations in packed red blood cells (PRBC) and platelets concentrates (PC) versus storage age. (DOC) [file pone.0033403.s007.doc]

***Table S5:*** *Spearman correlations of cytokine concentrations in packed red blood cells (PRBC) and platelets concentrates (PC) versus storage age.*

| **packed red blood cells (PRCP)** | | | |
| --- | --- | --- | --- |
| **mediator** | **r-value** | **p-value** | **n** |
| **IL-6** | n.d. | n.a. | n.a. |
| **IL-8** | n.d. | n.a. | n.a. |
| **TGF-β** | n.d. | n.a. | n.a. |
| **MCP-1** | 0.352 | **0.007 *** | 56 |
| **sCD40L** | 0.422 | **0.001 *** | 56 |
| **CXCL1** | 0.429 | **0.001 *** | 55 |
|  |  |  |  |
|  |  |  |  |
| **platelet concentrates (PC)** | | |  |
| **mediator** | **r-value** | **p-value** | **n** |
| **IL-6** | n.d. | n.a. | n.a. |
| **IL-8** | n.d. | n.a. | n.a. |
| **TGF- β** | -0.213 | 0.160 | 45 |
| **MCP-1** | 0.127 | 0.393 | 47 |
| **sCD40L** | -0.199 | 0.189 | 45 |
| **CXCL1** | n.d. | n.d. | n.a. |

IL-6: interleukin-6; IL-8: interleukin-8; TGF- β: transforming-growth-factor- β; MCP-1: monocyte chemoattractant protein-1; sCD40L: soluble CD40 ligand; CXCL1: chemokine ligand 1

n.d.: not detectable (cytokine concentrations below detection limit of the ELISA Kit)

n.a.: not applicable

* Correlation is significant at the 0.01 level (2-tailed)
