# Supplementary material for: Effects of Blood Products on Inflammatory Response in Endothelial Cells In Vitro
Source: PLoS One. 2012 Mar 16;7(3):e33403. doi: 10.1371/journal.pone.0033403 (PMC3306413; doi:10.1371/journal.pone.0033403)
Supplement: Table S6 — Spearman correlations of lipid concentration versus storage age in packed red blood cells (PRBC) and platelets concentrates (PC). (DOC) [file pone.0033403.s008.doc]

***Table S6:*** *Spearman correlations of lipid concentration versus storage age in packed red blood cells (PRBC) and platelets concentrates (PC).*

| **blood product** | **r-value** | **p-value** | **n** |
| --- | --- | --- | --- |
| PRBC | 0.457 | **0.043 *** | 56 |
| PC (pooled) | 0.672 | **0.004 **** | 25 |
| PC (apheresis) | 0.88 | **0.002 **** | 24 |

* Correlation is significant at the 0.05 level (2-tailed)

** Correlation is significant at the 0.01 level (2-tailed)
