# Supplementary material for: Effects of Blood Products on Inflammatory Response in Endothelial Cells In Vitro
Source: PLoS One. 2012 Mar 16;7(3):e33403. doi: 10.1371/journal.pone.0033403 (PMC3306413; doi:10.1371/journal.pone.0033403)
Supplement: Table S7 — Age-dependent assessment of neutrophil transmigration. (DOC) [file pone.0033403.s009.doc]

***Table S7:*** *Age-dependent assessment of neutrophil transmigration.*

|  | Unstandardized Coefficients | 95.0% Confidence Interval for B | | Standardized Coefficients | Sign. |
| --- | --- | --- | --- | --- | --- |
|  | B | Lower Bound | Upper Bound | Beta |  |
| Storage age | .023 | -.026 | .072 | .241 | .282 |
| Type of blood product | 1.815752289 | .986 | 2.646 | 1.125 | **0.002** |

R^2^: 0.914, N=20; dependent variable: transmigration of neutrophils.
