# Supplementary material for: Effects of Blood Products on Inflammatory Response in Endothelial Cells In Vitro
Source: PLoS One. 2012 Mar 16;7(3):e33403. doi: 10.1371/journal.pone.0033403 (PMC3306413; doi:10.1371/journal.pone.0033403)
Supplement: Table S8 — Influence of baseline sCD40L and lipid concentration in blood products on inflammatory mediator release in endothelial cells. (DOC) [file pone.0033403.s010.doc]

***Table S8.*** *Influence of baseline sCD40L and lipid concentration in blood products on inflammatory mediator release in endothelial cells.*

Inflammatory response in unstimulated endothelial cells

| Dependent variable | Independent variable | Standardized Coefficients | Unstandardized Coefficients | 95% Confidence Interval for B | | Sig. |
| --- | --- | --- | --- | --- | --- | --- |
|  |  | Beta | B | Lower Bound | Upper Bound |  |
| IL-6 | sCD40L | -.285 | -.003 | -.005 | -.001 | **<0.01** |
|  | LIPID | .447 | 24.984 | 14.519 | 35.450 | **<0.001** |
| IL-8 | sCD40L | -.370 | -.027 | -.040 | -.013 | **<0.001** |
|  | LIPID | .402 | 152.452 | 81.571 | 223.333 | **<0.001** |
| CXCL1 | sCD40L | -.369 | -.047 | -.071 | -.023 | **<0.001** |
|  | LIPID | .337 | 226.206 | 97.984 | 354.428 | **0.001** |

N=100. R^2^ 0.208 (IL-6), R^2^ 0.212 (IL-8), R^2^ 0.178 (CXCL1)

IL-6: interleukin-6; IL-8: interleukin-8; CXCL1: chemokine ligand 1; sCD40L: soluble CD40 ligand

Inflammatory response in stimulated endothelial cells

| Dependent variable | Independent variable | Standardized Coefficients | Unstandardized Coefficients | 95.0% Confidence Interval for B | | Sig. |
| --- | --- | --- | --- | --- | --- | --- |
|  |  | Beta | B | Lower Bound | Upper Bound |  |
| IL-6 | sCD40L | -.337 | -.528 | -.804 | -.251 | **<0.001** |
|  | LIPID | .538 | 4435.660 | 2981.724 | 5889.595 | **<0.001** |
| IL-8 | sCD40L | -.273 | -.564 | -.978 | -.150 | **0.008** |
|  | LIPID | .231 | 2510.874 | 330.475 | 4691.274 | **0.024** |
| CXCL-1 | sCD40L | -.329 | -1.614 | -2.545 | -.682 | **0.001** |
|  | LIPID | .389 | 10050.850 | 5145.640 | 14956.060 | **<0.001** |

N=100. R^2^ 0.298 (IL-6), R^2^ 0.092 (IL-8), R^2^ 0.186 (CXCL1)

IL-6: interleukin-6; IL-8: interleukin-8; CXCL1: chemokine ligand 1; sCD40L: soluble CD40 ligand
