# Supplementary material for: Effects of Blood Products on Inflammatory Response in Endothelial Cells In Vitro
Source: PLoS One. 2012 Mar 16;7(3):e33403. doi: 10.1371/journal.pone.0033403 (PMC3306413; doi:10.1371/journal.pone.0033403)
Supplement: Table S9 — Correlation of baseline sCD40L and lipid concentration in blood products with transendothelial migration of neutrophils. (DOC) [file pone.0033403.s011.doc]

***Table S9.*** *Correlation of baseline sCD40L and lipid concentration in blood products with transendothelial migration of neutrophils.*

|  | Standardized Coefficients | Unstandardized Coefficients | 95% Confidence Interval for B | | Sig. |
| --- | --- | --- | --- | --- | --- |
|  | Beta | B | Lower Bound | Upper Bound |  |
| sCD40L | .058 | .000 | .000 | .000 | .664 |
| Lipids | .675 | .249 | .165 | .334 | **<0.001** |
| LPS * sCD40L | -.061 | .000 | .000 | .000 | .673 |
| LPS * lipids | .058 | .020 | -.070 | .111 | .653 |

R^2^: 0.502 N: 62

sCD40L: soluble CD40 ligand; LPS: lipopolysaccharide
